# Bochdalek hernias associated with intrathoracic kidney: A case report and systematic review of outcomes including renal function 

**DOI:** 10.5414/CNCS109962

**Published:** 2020-01-28

**Authors:** Arunraj Navaratnarajah, Vaughn Barry, Rawya Charif

**Affiliations:** Renal Section, Department of Medicine, Hammersmith Hospital Campus, Imperial College London, Du Cane Road, London, UK

**Keywords:** bochdalek hernia, intrathoracic kidney

## Abstract

Background: An intrathoracic kidney is a very rare form of ectopic kidney. Though increasingly recognized in the literature, impact on renal function is less well described. We report the case of a 67-year-old South Asian gentleman who presented with intrathoracic kidney and chronic kidney disease. We carried out a systematic review of the available literature on intrathoracic kidney, in order to characterize the typical clinical features, and describe likely clinical course and possible renal and extra-renal complications associated with this form of ectopia. Materials and methods: A structured search using PubMed identified all relevant published case reports from 1988 to 2018, with search restricted to papers in English, and to adult cases only (> 18 years of age). 124 records were identified, and after screening for eligibility, 34 case reports were analyzed. Results: Median age was 53.5 years, with no gender predominance. 68% (27/34) of cases were symptomatic. 29% (10/34) had a significant complication associated with their intrathoracic kidney, with 3 cases with either documented chronic or end-stage kidney disease. 26% (9/34) required surgical intervention. Conclusion: Though previously regarded as a benign entity, results from our systematic review, bearing in mind susceptibility to publication bias, suggests an appreciable risk of symptoms, complications, and in the minority a risk to kidney function. We recommend close biochemical and imaging surveillance of affected patients, with low threshold for intervention in those with renovascular stenosis, reflux, or hydronephrosis.

## Introduction 

In 1848, Vincent Alexander Bochdalek first described a congenital diaphragmatic hernia, characterized by the passage of abdominal contents into the thoracic cavity [1]. Abdominal organs involved are usually the small bowel, spleen, stomach, colon, and left lobe of the liver. Intrathoracic herniation of kidneys, first described by Wolfromm in 1940 using retrograde pyelography [2], is very rare, with reported incidence of 0.25%, and accounts for just 5% of all renal ectopias [3]. 

In neonates, congenital diaphragmatic hernias can be associated with considerable morbidity and mortality [4], with possible pulmonary hypoplasia [5] or persistent fetal circulation [6], relying on early surgical correction. Later presentation in adults however is usually asymptomatic and discovery typically incidental on chest radiography. Males are more often affected, and the posterolateral diaphragmatic defect is usually on the left side in 80 – 90% [7]. 

We present a case of incidentally diagnosed Bochdalek hernia with right-sided intrathoracic kidney in an adult male as part of diagnostic work-up for chronic kidney disease (CKD). 

## Case presentation 

A 67-year-old gentleman, of Indian origin, was referred to our kidney clinic in view of decline in glomerular filtration rate (GFR). Comorbidities included hypertension, type II diabetes, asthma, and osteoarthritis. He was asymptomatic, denying breathlessness, arthralgia, macroscopic hematuria, and lower urinary tract symptoms. Glycemic control was good, with no reported neuropathy or retinopathy. There were no reported problems at birth, no history of abdominal trauma, and no family history of kidney disease or congenital anomalies. He denied taking non-steroidal anti-inflammatory drugs, over-the-counter preparations, and recreational drugs, and was a non-smoker. 

Physical examination was unremarkable with no evidence of pulmonary edema. Laboratory tests showed hemoglobin 11.5 g/dL, potassium 5.1 mmol/L, albumin 33 g/L, creatinine 133 µmol/L, and MDRD-calculated eGFR 47 mL/min. Creatinine in 2015 was 110 µmol/L. HbA1c was 48 mmol/mol. Urinalysis demonstrated trace proteinuria with no microscopic hematuria. Urinary albumin-creatinine ratio was subsequently 3.3 mg/mmol. Further analysis ruled out infectious disease (HIV, hepatitis B, and hepatitis C) and an autoimmune process (no autoantibodies, negative ANA, ANCA, GBM, normal serum complement). There was no evidence of a monoclonal band. 

**Figure 1.**
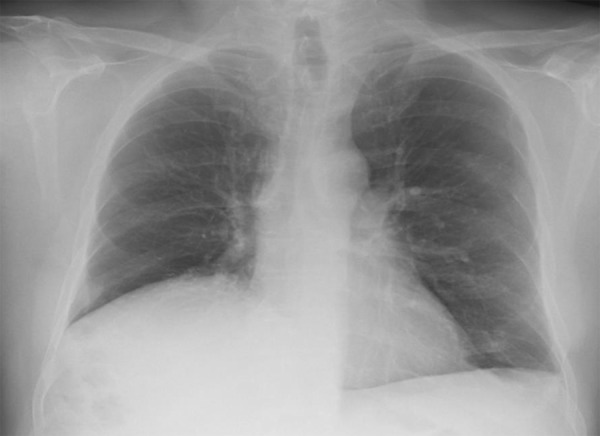
Chest radiograph demonstrating raised hemidiaphragm.

**Figure 2.**
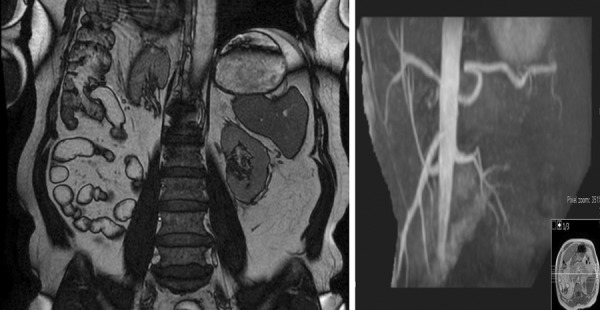
MRA demonstrating higher position of right kidney than left secondary to a right hemidiaphragmatic hernia containing the right lobe of liver, right kidney, and hepatic flexure. Right kidney 7.4 cm and left kidney 8.4 cm, with no evidence of renal artery stenosis.

**Figure 3.**
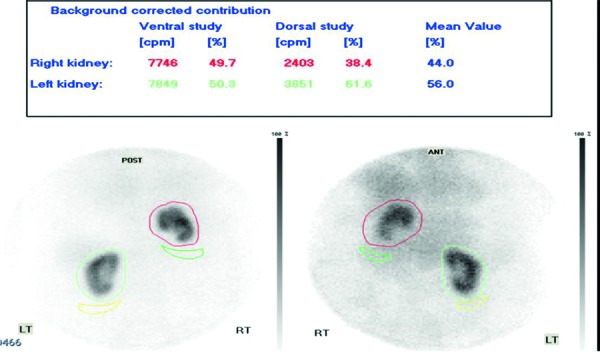
Renal DMSA scan demonstrating split function between right and left kidney (44% : 56%).

Historical chest radiographs (Figure 1) revealed an elevated right hemidiaphragm. An ultrasound of the urinary tract revealed right kidney 7.6 cm and left kidney 8.4 cm with preserved cortices. There were no calculi identified, with no features of pelvicalyceal dilatation. Prostate volume was 26 mL. Comment was made regarding limited views of the right kidney raising the possibility of an atrophic right kidney. Magnetic resonance angiography (MRA) (Figure 2) confirmed a smaller right kidney, however highlighted a right hemidiaphragmatic hernia containing the right lobe of the liver, right kidney, and hepatic flexure. Each kidney had single renal artery supply with no evidence of renal artery stenosis. Dimercaptosuccinic acid (DMSA) scan (Figure 3) revealed equal split function with no evidence of tracer hold-up, confirming unobstructed kidneys. 

Underlying renal diagnosis was unclear. He had stage III CKD without significant proteinuria, out of keep with a diagnosis of diabetic nephropathy. Macro-renovascular disease was excluded with MRA, however the possibility of small-vessel renal disease with ischemic nephropathy was possible together with age-related nephrosclerosis. Split-function with DMSA was equal with no features of obstruction, excluding vesicoureteric junction (VUJ) obstruction with intrathoracic renal ectopia. Given negative immunological and virology screen, he was not biopsied, and managed with cardiovascular and glycemic optimization. 

## Systematic review 

There is a paucity of data relating to long-term renal outcomes in such adults, with evidence restricted to anecdotal case reports. We systematically identified all available reported adult cases of intrathoracic kidney to describe clinical presentation, treatment, and renal outcomes to better inform responsible clinicians on the optimal management of this entity. 

Published studies (full-text, peer-reviewed) from 1988 to December 2018 relating to intrathoracic kidneys were found by utilizing a thorough search strategy of PubMed. The following search terms were used: intrathoracic kidney, thoracic kidney, and renal outcomes. Reference lists of chosen articles were searched to further identify relevant articles. 

Eligibility criteria for inclusion in the review was a specific focus on renal outcomes of intrathoracic kidneys within an adult population (> 18 years of age). Identified articles were assessed for inclusion independently by two authors. Reviews, commentaries, editorials, and non-English articles were excluded. 

Data extracted included: author, year, demographics of the patient sample, including age, proportion of males, clinical features, symptom duration prior to presentation, laterality of intrathoracic kidney, diagnostic work-up including imaging modality, renal function (laboratory data if available), treatment, and any reported complications. Given the qualitative, summative nature of this review, a meta-analysis was not possible and effect sizes could not be calculated. 

## Results 

**Figure 4.**
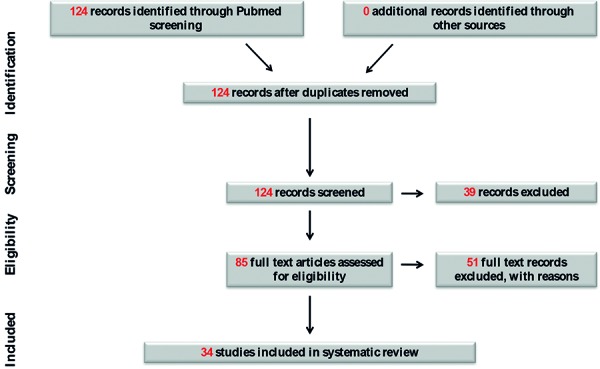
Intrathoracic kidneys and renal outcomes search strategy using PRISMA flowchart.

We examined 123 articles and identified 34 case reports relevant to intrathoracic kidneys in adults, reported from 1988 to 2018. The search strategy and flow diagram (Figure 4) are presented using PRISMA guidelines [8]. Case reports are summarized in Table 1. 17/34 (50%) cases were male. Median age was 53.5 years, with range 22 – 83 years. 11/34 (32%) cases were picked up as incidental findings with affected patients asymptomatic. In those with symptoms, 7/34 (32%) patients had symptoms related to the gastrointestinal tract (epigastric pain, nausea, vomiting, post-prandial pain), and 6/34 (18%) patients presented with chest pain. Other reported symptoms included respiratory complaints of cough or breathlessness (7/34, 21%), flank pain (4/34, 12%), and lower urinary tract symptoms (3/34, 9%). Incidental cases were often picked up by routine CXR, and characterized with CT or MR. Often ultrasound imaging reported either a smaller or missing kidney when focused on the abdomen. Incidental findings were also reported with echocardiography, IV urography, and myocardial scintigraphy. The majority of cases were right-sided (20/34, 59%). 

In 25/34 (74%) reported cases, renal function with serum creatinine or creatinine clearance was not described. 6 cases had documented normal renal function. 2 cases had evidence of moderate CKD: 1 patient with serum creatinine 140 µmol/L and the other patient with more advanced CKD with serum creatinine 229 µmol/L. The latter patient had a hydronephrotic left intrathoracic kidney with the contralateral kidney multi-loculated and cystic, whilst the former patient had a background of diabetes and hypertension. 1 case was established on maintenance hemodialysis, with Doppler evidence of renal artery stenosis affecting the intrathoracic kidney. Her end-stage kidney disease (ESKD) was presumed secondary to renovascular hypertension, and she required five antihypertensive agents for blood pressure control. 

Other reported complications included malignancy with clear cell renal cell carcinoma (1/34, 3%), renal calculi (2/34, 6%), pyelonephritis (1/34, 3%), acute respiratory failure (1/34, 3%), hydronephrosis (2/34, 6%), varicocele (1/34, 3%), and delayed perfusion relative to the contralateral intra-abdominal kidney (1/34, 3%). Intervention was required in few with surgical repair of diaphragmatic defect (4/34, 12%), partial nephrectomy (1/34, 3%), emergency laparotomy and right thoracotomy (1/34, 3%), percutaneous nephrolithotomy (2/34, 6%), and right-sided varicocelectomy (1/34, 3%). 

## Discussion 

The clinical case described raised some important questions. Firstly, are ectopic kidneys in the thorax incidental congenital variants of no clinical relevance? Secondly, what is the best imaging modality for detection and characterization? Thirdly, what complications may arise from intrathoracic kidneys, and with what frequency? And finally, what effect do they have on renal function, if any? This systematic review of previously published case reports addresses some of the gaps in understanding surrounding intrathoracic kidneys, and helps nephrologists and urologists in counselling affected patients on likely clinical course and renal prognosis. 

### Why do they occur? 

Intrathoracic kidneys are defined by partial or complete herniation of the kidney above the hemidiaphragm into the posterior mediastinal compartment of the thorax [9]. The definitive kidney derives from the metanephros and ascends up to reach its final position by the end of the 8^th^ week of intrauterine life. Aberrations in this complex, sequential developmental pathway predispose to ectopia [10]. Delayed closure or maldevelopment of the pleuroperitoneal membrane which separates the pleural cavity and peritoneal cavity usually by 8 weeks of intrauterine life, together with delayed ingrowths of the ureter bud into the metanephros, is one suggested mechanism. Attention has also focused on the interaction between the metanephros and mesonephros. The metanephros migrates cranially, and mesonephric tissue involutes in the opposite direction, with delayed involution of the mesonephric tissue resulting in extension of the renal tract, and thoracic ectopia [11]. 

### Demographics 

It is more common in infants, and rarely progresses to adulthood as the majority of diaphragmatic hernias present as neonatal respiratory distress or gastrointestinal obstruction, and mandate immediate surgical intervention. Reports of intrathoracic kidneys in adulthood are limited however. Donat et al. [12] identified in their review 131 cases where intrathoracic kidneys were described between 1922 and 1986, and highlighted reference to another 47 in the Japanese literature. Using PubMed, we found 33 relevant case reports of intrathoracic kidneys over the past 30 years. In Donat et al.’s [12] literature review, there was a male predominance with 63% male, though in our series there were an equal number of male and female cases affected. 

In our cohort, the majority of intrathoracic kidneys were right-sided. In previous case series, Bochdalek hernias were typically left-sided, in 61 – 90% of cases [12, 13]. A number of factors favor left-sided positioning [14, 15]: 1) earlier embryonic fusion of right-sided pleuroperitoneal folds, 2) left hemidiaphragm congenitally has a weaker structure than the right, and 3) liver on the right side serves as extra protection, with narrowing of the right pleuroperitoneal canal by the caudate lobe. It has been suggested that left-sided thoracic kidneys are more symptomatic [16]. Rarely, intrathoracic kidneys are bilateral (with a reported prevalence of 2% among thoracic ectopias), though none were described in our series, with recent reports restricted to the pediatric literature [17]. 

### Classification 

Pfister-Goedeke and Burnier [18] classified four types of intrathoracic kidney: 1) thoracic renal ectopia with eventration of diaphragm (commonest cause), 2) thoracic renal ectopia with diaphragmatic herniation, 3) traumatic rupture of diaphragm with renal ectopia, and 4) thoracic renal ectopia with closed diaphragm. As well as congenital, kidney herniation can also be acquired, with direct migration of the kidney through t he diaphragmatic defect during the patient’s lifetime. 

### Radiology 

CXR findings often reflect a posterior mediastinal shadow, with a rounded mass behind the cardiac silhouette and elevation of the affected hemidiaphragm [19]. CT provides characterization and anatomical definition of the lesion and helps in differentiating renal ectopia from causes for a posterior mediastinal lesion on chest radiograph, including pulmonary sequestration and neurogenic masses. Abdominal ultrasound reports often describe an atrophic or missing kidney, so in such cases, follow-up chest imaging to avoid misdiagnosis is advised. Renal scintigraphy with DMSA provides an estimate of split renal function and can exclude reflux and obstruction [20]. 

In the majority of cases, the intrathoracic kidney is located in the thoracic cavity and not in the pleural space, with renal vessels and ureter passing through the foramen of Bochdalek. Commensurate herniation of abdominal viscera is common. Usually, there is no contralateral hypertrophy. Rarely, intrathoracic kidneys have been reported to possess congenital anomalies such as Pelviureteric junction (PUJ) obstruction, malpositioning and duplication of the renal pelvis and ureters, though this was not described in our series. 

Typical radiological features include rotational anomaly, long ureter, anomalous high derivation of the renal vessel, and medial deviation of the lower pole of the kidney [21]. Absence of such features points to an acquired etiology rather than congenital intrathoracic renal ectopia. Almost all cases are posteriorly located in the thorax. Those cases that have been described with anterior location may relate to diaphragmatic agenesis rather than traditional Bochdalek hernias or eventration [22] 

### Clinical presentation and complications 

Symptoms from intrathoracic kidneys ranged from respiratory, gastrointestinal, and genitourinary complaints. Almost a quarter of the reported cases were associated with a complication. Traditionally, inferior ectopic kidneys are more prone to infection, obstruction, and stone formation. 1 case reported pyelonephritis with secondary *E. coli* bacteremia requiring 2 weeks of antibiotics [23]. The stretched ureter in intrathoracic kidneys usually provides good drainage, however 2 cases in our series had hydronephrosis [24, 25]. A further 2 cases developed renal calculi requiring percutaneous nephrolithotomy [26, 27]. An important practical point, ureteroscopy and stone extraction was not feasible given intrathoracic location, hence the need for percutaneous intervention. Though remarkably rare, there should be an awareness of the possibility of acute decline in patients with hernias associated with intrathoracic kidney. 1 case was in extremis with acute respiratory failure secondary to lung compression in the context of herniation of a strangulated colon and right kidney [28]. There were no reported cases of viscus perforation or hemorrhage. 

Vascular complications have been described previously in patients with renal ectopia [29] and include entrapment of the renal artery by the diaphragmatic crura, possession of multiple aberrant arteries, to more typical plaque-mediated vascular stenosis [30]. In our series, 1 case described a relative perfusion delay to the contralateral kidney likely secondary to traction phenomena on the vascular pedicle [25], and in 1 case, impaired venous return predisposed to a varicocele [31]. Our systematic review also included the first published case report of renal artery stenosis to an intrathoracic kidney with resultant ESKD [30]. Early intervention in this case with either angioplasty/stenting or even nephrectomy (intrathoracic kidney) may have improved blood pressure control, stabilizing kidney function. This highlights a key role for MR or CT angiography to clearly delineate the vascular supply of such ectopic organs and a low threshold for intervention if stenosis identified. 

### Effect on renal function 

In our case, it was difficult to determine the etiology of the patient’s renal insufficiency, and the comorbid diagnoses of diabetes and hypertension were likely culprits rather than the ectopic location of his right kidney. Evidence in support of this is the equal split function and also the stability in radiographic appearances over the years. Renal function was documented in only a quarter of cases included in our review. As discussed, 1 case reached ESKD in the context of renovascular hypertension secondary to stenosis of the artery supplying the intrathoracic kidney. Of the 2 cases with reported CKD, 1 had hydronephrosis of the thoracic kidney and a multicystic contralateral kidney [25], and the other case may have been attributable to underlying diabetes and hypertension [20]. 

Surgical correction of hernias associated with intrathoracic kidneys have been described (Table 1), with no significant complications reported. Compared to open approaches, minimally invasive laparoscopic and thoracoscopic diaphragmatic hernia repairs have been associated with favorable outcomes. Robotic-assisted surgery promises to optimize this field further [24]. The case described earlier with acute respiratory compromise required emergent laparotomy and right thoracotomy. With this in mind, some favor pre-emptive elective surgical repairs on larger hernias, because of the risk of potential complications of bowel strangulation and incarceration. Other than size, it is difficult to predict which hernias require correction. 

### Limitations 

A major weakness of the study is that large numbers of the included case reports did not document renal function, therefore it is difficult to draw meaningful conclusions on the incidence of renal dysfunction with intrathoracic kidneys. It is likely that in the cases in which renal function was not reported, renal function was normal. Furthermore, caution must be taken in over-interpreting our surprisingly high frequency of associated symptoms, given vulnerability to positive-outcome publication bias and a tendency to report on the unusual. Outcomes of missing or non-reported cases might have been very different. Despite the shortcomings associated with systematic review of retrospective case reports, it was necessary to do so, in view of the rarity of intrathoracic kidneys together with absence of other research designs in this field. 

## Conclusion 

Concluding on the effect of intrathoracic kidneys on renal outcomes is clearly limited by the available literature, primarily case reports, many of which lack data on renal function. This review does highlight that though intrathoracic kidneys are potentially associated with a myriad of symptoms, the majority (75% of cases reviewed) are without complication, with prognosis unaffected. A “watch and wait strategy” can be applied to such patients. Serial measures of kidney function and imaging surveillance are indicated. In some cases, particularly acquired hernias, there can often be progression in the contents of the hernia sac, and secondary enlargement with clinical effect [24]. Though rare, malignancy affecting the thoracic kidney has also been reported [32], again providing further indication for careful surveillance. Early intervention either radiologically or surgically should be considered if complications of renovascular stenosis, VUJ reflux or obstruction arise, with better outcomes when planned electively. 

## Funding 

None. 

## Conflict of interest 

None declared. 

**Table 1.** Case reports of intrathoracic kidneys in adults.

Author	Journal	Year of publication	Country	Age/gender	Clinical presentation	Initial imaging	Location	Renal function	PC dilatation	Complication	Intervention	Additional comment
Selene et al. [33]	Colorectal disease	2018	Italy	67 F	Epigastric pain	CT	R	Unknown	–		Laparoscopic repair of congenital diaphragmatic hernia	
Dell’Atti et al. [31]	Arch Ital Urol Androl	2017	Italy	22 M	Chronic scrotal pain	Not seen on USS, seen on CT and MRA	R	Unknown	Non-dilated	R Varicocele	R varicocolectomy with microsurgical inguinal approach	
Sharma et al. [32]	Images in Clinical Urology	2016	USA	55 F	Asymptomatic	CT	R	Normal renal function	–	Clear cell renal cell carcinoma	Open right partial nephrectomy (diaphragmatic defect not repaired)	
Natarajan et al. [20]	Indian Journal of Nuclear Medicine	2016	India	62 M	Asymptomatic	L kidney not seen on US, so sent for DMSA (adequate cortical function and non-obstructed drainage)	L	Cr 140	–			Diabetic and hypertensive
Chen et al. [24]	Journal of Robotic Surgery	2015	USA	80 F	Chronic epigastric pain, nausea, occasional dyspnoea	CT	R	Normal renal function	Hydronephrotic		Robotic-assisted repair	Progression in size since historical scan (4 years), at which point omentum only in hernia
Yong-sun Noh et al. [23]	Korean J Internal Medicine	2015	Korea	56 F	Fever, epigastric pain, costochondral tenderness	CXR	L	Unknown	–	Acute pyelonephritis		No VUJ reflux
Niwa et al. [34]	Japanese Society of Internal Medicine	2014	Japan	28 M	Asymptomatic	CXR	L	Unknown	–			
Onuk et al. [35]	Urologia Internationalis	2014	Turkey	72 F	Symptoms NOS	CXR	R	Unknown	–			
Jha et al. [22]	Hernia	2014	India	25 F	Occasional dyspnoea/chest pain	CXR/USS	R	Unknown	–		Surgical repair of diaphragmatic defect	
Clarkson et al. [9]	British Journal of Radiology	2014	UK	76 F	Lower urinary tract symptoms	CT	R	Unknown	–			
Gupta et al. [36]	Hong Kong Journal of Nephrology	2013	India	20 F	Asymptomatic	CXR	R	Normal renal function	Non-dilated			
Ekrikpo et al. [30]	Case Reports in Nephrology	2013	Nigeria	38 F	Dyspnoea/Leg swelling	USS	L	Cr 900 (pre-maintenance HD)	Non-dilated	Left renal artery stenosis		Both kidneys scarred
Bianchi et al. [13]	Journal of Medical Case Reports	2013	Italy	57 W	Asymptomatic	CT	R	Unknown	–			
Pandey et al. [37]	BMJ Case Reports	2012	India	62 M	Lower urinary tract symptoms	USS	R	Unknown	Non-dilated			
Darwazah et al [38]	Journal of Cardiac Surgery	2011	Israel	48 M	Asymptomatic	CT	L	Cr 80-100	Non-dilated			
Ahmed AH et al. [39]	Mymensingh Med J	2011	Bangladesh	52 M	(Left) Chest pain	CXR/USS	L	Unknown	–			
Fiaschetti et al. [25]	Case Reports in Medicine	2010	Italy	62 M	Cough, post- prandial pain, difficulty with micturition	CXR	L	Cr 229.8	Hydronephrotic	Perfusion delay to left kidney observed due to traction phenomena of vascular pedicle		
Shi-Dong Chung et al. [40]	Kidney International	2010	Taiwan	55 M	Epigtastric pain	CT	R	Unknown	–			
Wei-Ning et al. [41, 42]	Clinical Nuclear Medicine	2010	Taiwan	83 M	Atypical Chest pain	Myocardial scintigraphy	R	Unknown	–			
Fadaii et al. [42]	Iranian Journal of Kidney Diseases	2008	Iran	72 F	Chest pain	CT	L	Cr 53	Non-dilated			
Subramanian et al. [43]	Urology	2008	USA	23 F	(Right) Flank pain	MR	R	Unknown	Non-dilated			
Singh et al. [27]	International Journal of Urology	2007	India	40 M	Chronic flank pain	CT	L	Unknown	Compact PC system’	Renal calculus	Percutaneous nephrolithotomy	
Lee et al. [44]	International Journal of Cardiology	2006	Taiwan	28 M	Chest pain	Echocardiogram	R	Unknown	–			
Oon et al. [45]	J Formos Med Association	2005	Taiwan	50 M	Chronic chest pain	CXR	L	Unknown	–			
Lenz et al. [26]	Urology	2003	USA	35 F	(Right) Flank pain, vomiting	IVU	R	Cr 61	Non-dilated	Renal calculus	Percutaneous nephrolithotomy	
Kanazawa et al. [28]	Surgery Today	2002	Japan	63 F	Acute dyspnoea	CXR	R	Unknown	–	Acute respiratory failure	Emergency laparotomy and right thoracotomy, direct closure of hernia opening	
Sidhu et al. [46]	Urologia Internationalis	2001	India	28 F	Intermittent cough	IVU	R	Unknown	–			
Jefferson et al. [47]	The Journal of Urology	2001	UK	30 M	Chest pain/exertional dyspnoea	CXR	L	Unknown	Non-dilated			
Yalcinbas et al. [48]	The Annals of Thoracic Surgery	2001	Turkey	21 M	Asymptomatic	CXR	L	Unknown	Non-dilated			
Toba et al. [49]	Clinical Nuclear Medicine	2000	Japan	66 M	Asymptomatic	Stress myocardial imaging then MAG-3	R	Unknown	–			
Kageyama et al. [50]	Images in Clinical Urology	2000	Japan	50 F	Asymptomatic	CXR	L	Unknown	Non-dilated		Likely acquired diaphragmatic hernia	
Suarez et al. [51]	Acquired intrathoracic kidney	1998	Mexico	63 F	Asymptomatic	CXR	R	Unknown	–			
Panossian et al. [52]	Chest	1995	USA	70 M	Asymptomatic	CXR	L	Unknown	–			
Donat et al. [12]	The Journal of Urology	1988	USA	42 M	(Left) lower quadrant pain	IVP	R	Unknown	–			
